# Clinician-led improvement in cancer care (CLICC) - testing a multifaceted implementation strategy to increase evidence-based prostate cancer care: phased randomised controlled trial - study protocol

**DOI:** 10.1186/1748-5908-9-64

**Published:** 2014-05-29

**Authors:** Bernadette (Bea) Brown, Jane Young, David P Smith, Andrew B Kneebone, Andrew J Brooks, Miranda Xhilaga, Amanda Dominello, Dianne L O’Connell, Mary Haines

**Affiliations:** 1 Sax Institute, Haymarket, Australia; 2 School of Public Health, University of Sydney, Camperdown, Australia; 3 Cancer Research Division, Cancer Council NSW, Sydney, Australia; 4 Griffith Health Institute, Griffith University, Gold Coast, QLD, Australia; 5 Department of Radiation Oncology, Royal North Shore Hospital, Sydney, Australia; 6 Northern Clinical School, University of Sydney, Camperdown, Australia; 7 NSW Agency for Clinical Innovation, Sydney, Australia; 8 Westmead Private Hospital, Westmead, Australia; 9 Westmead Clinical School, University of Sydney, Camperdown, Australia; 10 Prostate Cancer Foundation of Australia, Melbourne, Australia; 11 School of Public Health and Community Medicine, University of New South Wales, Sydney, Australia; 12 School of Medicine and Public Health, University of Newcastle, Callaghan, NSW, Australia

**Keywords:** Implementation strategies, Clinical practice guidelines, Clinical networks, Cancer, Interventions

## Abstract

**Background:**

Clinical practice guidelines have been widely developed and disseminated with the aim of improving healthcare processes and patient outcomes but the uptake of evidence-based practice remains haphazard. There is a need to develop effective implementation methods to achieve large-scale adoption of proven innovations and recommended care. Clinical networks are increasingly being viewed as a vehicle through which evidence-based care can be embedded into healthcare systems using a collegial approach to agree on and implement a range of strategies within hospitals. In Australia, the provision of evidence-based care for men with prostate cancer has been identified as a high priority. Clinical audits have shown that fewer than 10% of patients in New South Wales (NSW) Australia at high risk of recurrence after radical prostatectomy receive guideline recommended radiation treatment following surgery. This trial will test a clinical network-based intervention to improve uptake of guideline recommended care for men with high-risk prostate cancer.

**Methods/Design:**

In Phase I, a phased randomised cluster trial will test a multifaceted intervention that harnesses the NSW Agency for Clinical Innovation (ACI) Urology Clinical Network to increase evidence-based care for men with high-risk prostate cancer following surgery. The intervention will be introduced in nine NSW hospitals over 10 months using a stepped wedge design. Outcome data (referral to radiation oncology for discussion of adjuvant radiotherapy in line with guideline recommended care or referral to a clinical trial of adjuvant versus salvage radiotherapy) will be collected through review of patient medical records. In Phase II, mixed methods will be used to identify mechanisms of provider and organisational change. Clinicians’ knowledge and attitudes will be assessed through surveys. Process outcome measures will be assessed through document review. Semi-structured interviews will be conducted to elucidate mechanisms of change.

**Discussion:**

The study will be one of the first randomised controlled trials to test the effectiveness of clinical networks to lead changes in clinical practice in hospitals treating patients with high-risk cancer. It will additionally provide direction regarding implementation strategies that can be effectively employed to encourage widespread adoption of clinical practice guidelines.

**Trial registration:**

Australian New Zealand Clinical Trials Registry (ANZCTR): ACTRN12611001251910.

## Background

### The evidence-practice gap

The discrepancy between research evidence and clinical practice is well documented [1], and remains one of the most persistent problems in providing high-quality healthcare [2]. Clinical practice guidelines have been extensively developed as a means to disseminate best practice and ensure clinical decision-making is informed by recent, credible research evidence, thereby improving healthcare processes and outcomes. However, timely and effective implementation of guidelines into clinical practice is inconsistent [3], and it remains surprisingly difficult to make changes across the health system even when there is compelling evidence [4].

The difficulty in achieving large scale adoption of proven innovations and recommended care (as well as discontinuing ineffective or harmful practices) has been characterised as a ‘translation block’ [5-8].

### Effective implementation

Previous research indicates that successful implementation of evidence-based care depends critically on the extent to which strategies address prospectively identified barriers, through theoretical frameworks of behaviour change [9,10], and promote provider acceptance [3]. Recommendations from clinical guidelines are more likely to become embedded within practice when they: are initiated and led by local clinical leaders; are tailored to the local context; and engage clinicians in the design of the implementation strategy [1,3,11-13]. Grol [14] argues that to effectively implement evidence-based practice, research urgently has to change so that it develops through collaborations between clinicians, researchers, patients, policy makers, and quality improvement experts.

Specifically, the growing body of evidence suggests several core implementation strategies are effective in bringing about system-wide and sustained change [1,11,15,16]:

1. Clinical champions/leaders supporting change within their practices and settings;

2. System, structural, and organisational support for system-wide changes to enable implementation strategies to be rolled out and scaled up (*e.g.*, legislation, resources, mechanisms for communication and collaboration between health sectors);

3. Ongoing monitoring, evaluation, and feedback of changes as they are implemented.

### Clinical networks—a medium for implementation

In New South Wales (NSW), Australia, a coordinated program of 30 clinical networks, institutes and taskforces has been established by the NSW Agency for Clinical Innovation (ACI), a board-governed statutory organisation funded by the NSW Ministry of Health.

These clinical networks of volunteer health professionals provide a framework for doctors, nurses, allied health professionals, managers, and consumers to collaborate across regional and service boundaries to drive improvements in service delivery and care outcomes through innovation in clinical practice.

This type of non-mandatory clinical network is increasingly being viewed as a vehicle through which evidence-based care can be embedded into healthcare systems using a collegial approach to agree on and implement a range of strategies within hospitals. They provide ‘bottom up’ views on the best ways of tackling complex healthcare problems coupled with the strategic and operational ‘top down’ support necessary to facilitate and champion changes in practice at the clinical interface [17,18]. There is evidence from ‘before and after’ controlled studies that when clinical practice guidelines are implemented through clinical networks there are improvements in compliance with guideline recommendations and the quality of care [19,20].

Clinical networks embody, or have the potential to enable, the core features of successful implementation strategies and therefore are a mechanism for health system change and increasing the uptake of evidence-based care for three reasons:

1. Clinical networks contain clinical leaders who can design and champion change to improve care within their practices and influence wider culture change within their healthcare settings.

2. Clinical networks are a ‘ready-made’ organisational structure through which innovations may be promulgated and accelerated by clinicians.

3. Clinical networks provide a structure to monitor and evaluate changes as they are implemented to answer questions about effectiveness and the success of implementation strategies.

### Prostate cancer clinical practice guidelines—an opportunity to translate research into effective healthcare practice

Prostate cancer is the most common cancer registered in Australia and is the second highest cause of cancer death in males [21]. Radical prostatectomy is the most frequent procedure for localised prostate cancer, however following surgery it is estimated that 20% to 50% of men are at ‘high risk’ of experiencing progression or recurrence [22-25]. A national strategy to improve prostate cancer services and thereby improve patients’ quality of life and survival identified the provision of evidence-based care for these men as a high priority [26]. Persuasive evidence from randomised controlled trials indicates the need to alter current practice by offering radiotherapy to men with adverse disease features following surgery as radiotherapy treatment halves the risk of recurrence [27-29] and improves biochemical disease-free survival [30]. A Grade B recommendation (denoting that the Clinical Practice Guideline expert working group considered that the body of evidence can be trusted to guide practice in most situations) in the *Clinical Practice Guidelines for the Management of Locally Advanced and Metastatic Prostate Cancer* produced by the Australian Cancer Network [31] recommends that ‘patients with extracapsular extension, seminal vesicle involvement or positive surgical margins receive post-operative external beam radiation therapy within four months of surgery.’ This recommendation is echoed in the more recently published American Urological Association Guideline, *Adjuvant and Salvage Radiotherapy after Prostatectomy*, which states ‘Physicians should offer adjuvant radiotherapy to patients with adverse pathologic findings at prostatectomy (Standard; Evidence Strength: Grade A)’ [32]. The most recently available data indicate less than 10% of patients with locally advanced prostate cancer in NSW Australia receive guideline recommended care [33]. Patterns of care for prostate cancer in NSW generally reflect practice in other Australian jurisdictions [34,35]. These data are consistent with that from the United States where less than 20% of eligible patients receive adjuvant radiotherapy, indicating substantial room for improvement [36]. Current evidence about strategies to encourage the adoption of clinical practice guidelines is limited [1-3,9,37] and provides little clear direction about approaches that can be effectively employed in specific settings.

### Aims

The aim of this study is to develop and trial a locally tailored, multifaceted implementation strategy that harnesses the NSW Agency for Clinical Innovation (ACI) Urology Clinical Network to increase evidence-based care for men with high-risk prostate cancer following prostatectomy in selected NSW hospitals [31]. Specifically, the aim is to increase referral to radiation oncology for a discussion about radiotherapy, and the associated risks and benefits of treatment, to support fully informed decision making.

An additional aim is to identify reasons why changes in behaviour and outcomes occurred or did not occur in study hospitals and why the implementation strategy did or did not result in increased compliance with guideline recommended care.

If the intervention is successful we will also assess the sustainability of increases in referral patterns within the hospitals through interviews with key stakeholders.

### Approach to intervention design

Any reason for resisting new practice is a barrier to change and the potential importance of such barriers and their influence on quality improvement activities has been highlighted in numerous studies [38-41]. A recent systematic review indicates that tailored interventions are more effective when they are designed to address prospectively identified local barriers to change [10]. A key component of our method is to tailor our intervention so that it incorporates features that will facilitate changes in provider behaviour by addressing local level obstacles.

**Figure 1:**
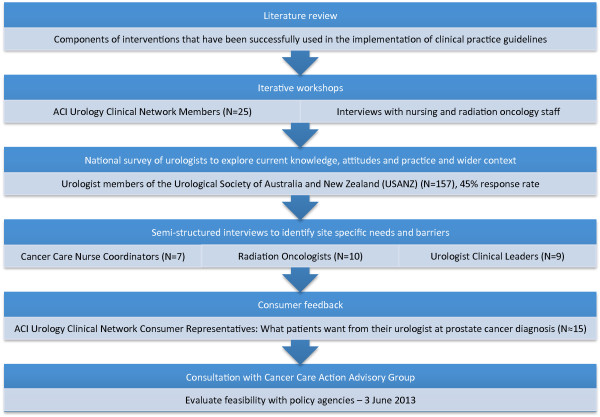
Approach to intervention design.

Intervention elements have been informed by reviews of the clinical practice change literature [9,11,37,38,42-61], and refined and tailored to take account of the organisational context in which providers practice through a multi-component needs and barriers analysis, including: iterative workshops with members of the ACI Urology Clinical Network; a national baseline survey (offered in web-based and paper form) of all urologist members of the Urological Society of Australia and New Zealand, the peak professional body, to explore current knowledge, attitudes and practice in the wider context (results published elsewhere); semi-structured interviews with urology, radiation oncology, and nursing staff at target hospitals to explore site specific practice and barriers; consumer feedback on what information patients want from their urologist; and consultation with a cancer policy advisory group to ensure intervention elements are feasible, scalable and potentially translatable to other cancers (see Figure 1 for summary).

Results from these activities indicate that, in priority order, barriers can be grouped into three main clusters:

1. Clinician: attitudes and beliefs held by individual clinicians about the validity of the evidence base supporting the guideline recommendation (54% of urologists surveyed agreed that the recommendation is based on a valid interpretation of the underlying evidence) - notably due to ongoing clinical trials, which raise doubts as to the treatment benefit of adjuvant radiotherapy versus early salvage radiotherapy; concerns about overtreatment and toxicity/side effects associated with radiotherapy and lack of familiarity with current radiotherapy techniques (two thirds of urologists surveyed agreed that patients may experience unnecessary discomfort if they follow the recommendation).

2. Patient: treatment preferences (perceived to be influenced by interaction with urologists).

3. Hospital system and processes: variation in urologists’ engagement with the multidisciplinary team (MDT) of specialist surgeons, medical oncologists, radiation oncologists, nurses and other allied health professionals providing specialist cancer care; and selective presentation of high-risk prostate cancer cases to the MDT resulting in inconsistent multidisciplinary discussion of all available treatment options and pathways.

### Conceptual model

**Figure 2:**
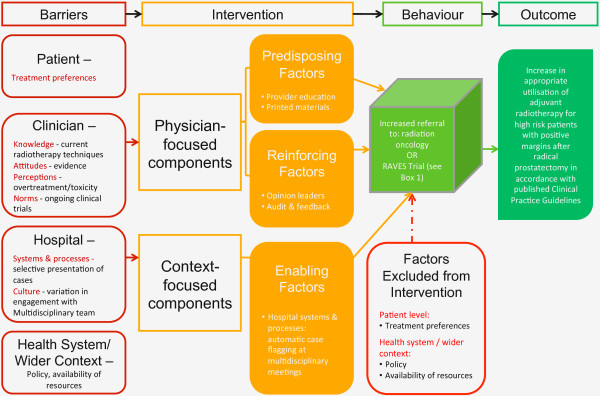
Conceptual model: adaptation of PRECEDE-PROCEED model of behaviour change.

Intervention components are underpinned by the PRECEDE-PROCEED theory of behaviour change [62,63] that relates interpersonal factors and system characteristics into one model to inform change in practice. This theory enables the integration of barriers identified through our mixed methods needs and barriers analysis into ‘predisposing factors’ (*e.g.*, knowledge and attitudes of the target group); ‘reinforcing factors’ (*e.g.*, opinions and behaviour of peers); and ‘enabling factors’ (*e.g.*, capacity of the system and hospital processes). This is one of the most widely used theories to support rigorous trials of the implementation of guidelines [16] and systematic reviews have shown that trials that intervene to alter these three factors are the most successful [13]. Figure 2 illustrates how the identified barriers to change in prostate cancer care have been grouped into the factors of the PRECEDE-PROCEED theory. Additionally, Figure 2 illustrates the intervention components that have been designed to target each barrier.

### Intervention components

#### Physician-focused components

1. Provider education (predisposing factor): The Urologist Clinical Leader at each hospital will be supported to facilitate an interactive education session at a routinely scheduled multidisciplinary team (MDT) meeting. This session will be moderated by members of the research team to ensure fidelity and will last approximately 10 to 15 minutes. Participants will be presented with an introduction to the study, including a summary of the evidence underlying the guideline recommendation through a video presentation to control for inconsistency across sites. The video includes the Co-Chair of the ACI Urology Clinical Network, a peer-identified national urologist opinion leader, and a consumer who introduce key messages through discussion of their practice and experience.

2. Dissemination of printed materials (predisposing factor): In the active implementation phase all urologists will be given a full copy of the *Clinical Practice Guidelines for the Management of Locally Advanced and Metastatic Prostate Cancer* and a summary card that allows quick reference to the evidence supporting the specific recommendation that is the focus of the study, together with information on potential side effects and toxicity. The reverse of this summary card provides information on current radiotherapy techniques and key points to guide impartial discussion with patients before and after surgery to support fully informed decision-making. This includes the potential need for multidisciplinary care and consultation with a radiation oncologist to obtain information about what radiotherapy would involve and the likely benefits and risks of treatment if high-risk features are found upon histopathological examination of the prostate specimen.

3. Opinion leaders (reinforcing factor): A key aspect of the intervention will be the use of Urologist Clinical Leaders in each hospital, identified by peers as being educationally influential, to engage the target group. Clinical Leaders will reinforce key messages, persuade peers to participate in the study and will model targeted referral behaviours and promote practice change [64]. Following the education session, Clinical Leaders will provide ongoing peer support and engage in discussions with colleagues to seek and provide feedback on practice and any continuing barriers to change. The Clinical Leaders are members of the ACI Urology Clinical Network and were recruited by the Network Co-Chair, an expert opinion leader who is influential due to his authority and status amongst his peers [65]. The introduction of key messages by a national opinion leader in the video presented at the education session provides an additional level of peer-to-peer influence.

4. Audit and feedback (reinforcing factor): Following commencement of the intervention, urologists will be provided with ongoing feedback reports detailing the number of patients referred to radiation oncology, at the individual, hospital and study level, obtained through data extraction from medical records. The feedback report will also include information on the number of patients at high risk who are discussed at MDT meetings. The initial feedback report will include baseline data. Feedback will be provided via email or SMS depending on the preferred method of communication of each participant. Aggregated quarterly feedback reports will additionally be presented verbally by the Clinical Leader at MDT meetings.

### Context-focused components

Guideline dissemination and educational components will address gaps in provider knowledge. However, a number of reviews indicate that increased knowledge is necessary but insufficient to change individual or organisational behaviour [41]. It is also necessary to enable change by increasing means or reducing barriers [66]. Therefore, in conjunction with physician-focused components, utilising the leverage of the ACI Urology Clinical Network to address the systems barriers identified through the mixed methods needs and barriers analysis, context-focused components will include a new system for automatic case flagging at MDT meetings (enabling factor). Urologists practising at the nine target hospitals will be requested to provide consent for the names of all patients who have had a histopathological examination of a radical prostatectomy specimen and who have extracapsular extension, positive surgical margins or seminal vesicle involvement to be submitted automatically to the hospital urology MDT meeting for discussion. Pathology providers will provide a list of all eligible patients to the MDT coordinator. This will reduce variation in practice and selective presentation of cases to the MDT meeting with the intent to promote more collaborative decision-making and increased referral to radiation oncology for high-risk patients.

## Methods

### Phase I: intervention rollout and implementation trial

#### Hypotheses

Compared with pre-intervention, a larger proportion of post-operative radical prostatectomy patients who are at high risk of recurrence (have extracapsular extension, seminal vesicle involvement or positive surgical margins) treated in hospitals after implementation of the intervention will receive a referral to radiation oncology for consideration of adjuvant radiotherapy or referral to the RAVES trial [Radiotherapy Adjuvant Vs Early Salvage (Protocol Number: TROG.08.03); see the ‘RAVES Trial’ subsection for details].

### Design

**Figure 3:**
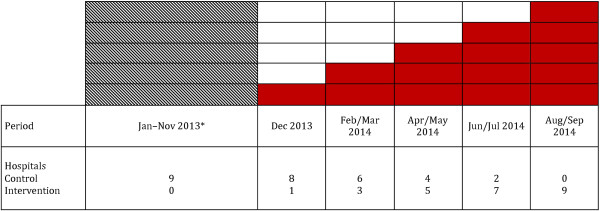
**Stepped wedge study design: staged rollout of intervention from December 2013 to September 2014.** The solid shaded blocks represent introduction of the intervention over 5 steps. The intervention will be rolled out across the nine hospitals in two-month blocks. Patient medical records will be reviewed for a period of 12 months following the interactive education session. Therefore data collection will not be completed until September 2015. *Control-only monitoring not part of the intervention study.

This will be a phased randomised cluster trial with phased introduction of a clinical network led organisational intervention in nine hospitals over 10 months. The order in which hospitals will receive the intervention will be determined randomly using a stepped wedge study design (see Figure 3). This design, originally developed for community studies, has more recently been applied to health service interventions in hospitals [67] and has the following advantages: provides a control comparison where geographic controls are not possible; allows all hospitals in the clinical network with multidisciplinary teams to take part in the intervention; enables the intervention to be tested within the parameters of real-world allocation of clinical network resources with a phased roll out of the hospital-based intervention; and complies with the Cochrane Effective Practice and Organisation of Care Group’s consensus statement about study designs of sufficient quality to be included in systematic reviews. This study will be conducted and reported in accordance with the CONSORT statement for the reporting of pragmatic trials [68,69].The intervention will be rolled out across the nine hospitals in five steps of two-month blocks from December 2013 to September 2014. Throughout the study, hospitals will either be in the active implementation (intervention) or passive (control) phase (see Figure 3). Eligibility criteria for inclusion are public hospitals: with a urology multidisciplinary team (MDT) comprising specialists, nurses, and allied health professionals; and that are members of the ACI Urology Clinical Network and have a urologist who will act as the Clinical Leader for that site. All urologists who are members of the urology multidisciplinary team at intervention hospitals will be eligible for inclusion (n ≈ 4 – 10 urologists per hospital).

### Outcomes

Primary outcomes are patient referral to radiation oncology for discussion of adjuvant radiotherapy in line with guideline recommended care or referral to the RAVES trial (see the ‘RAVES Trial’ subsection for details). Secondary outcomes include: an initial patient consultation with a radiation oncologist; enrolment in the RAVES trial; and commencement of radiotherapy.

### RAVES Trial – an opportunity to demonstrate shift in equipoise

RAVES [Radiotherapy Adjuvant Vs Early Salvage (Protocol Number: TROG.08.03)] is a multi-centre phase III clinical trial comparing survival and quality of life outcomes for patients at high-risk post prostatectomy who are randomised to have: i) radiotherapy deferred (salvage radiotherapy) until their prostate specific antigen (PSA) begins to rise (common current practice); OR ii) immediate radiotherapy (adjuvant radiotherapy) after surgery (regarded as evidence-based standard of care). This is seen as a very important local trial as, despite international evidence that adjuvant radiotherapy is effective, this practice has not been widely adopted due to Urologists’ concerns about side effects and overtreatment. The aim of the RAVES trial is to determine whether salvage radiotherapy is as effective as adjuvant radiotherapy and results in improved quality of life.

### Data collection—data extraction from patients’ medical records

Outcome data to assess changes in healthcare practice will be collected through data extraction from urologists’ and radiotherapy patients’ medical records by independent, trained research assistants who are blind to the date that the intervention was commenced at the hospital. Baseline data will be collected retrospectively for patients undergoing a radical prostatectomy during January 2013 to November 2013. Pilot testing of the medical record review tools and processes will allow us to train the research assistants and establish and test data collection procedures.

### Information from medical records

Treatment outcomes that will be collected through medical record review for cases with extracapsular extension, seminal vesicle involvement or positive surgical margins (confirmed by pathology reports) are: referral to radiotherapy, taken from the surgeon’s notes (including dates of surgery and referral) or in the case where there was no referral that radiotherapy was discussed and the reason(s) for not referring to radiotherapy; uptake of radiotherapy or enrolment into the RAVES trial from the radiation oncology database; and time between surgery and commencement of radiotherapy. Individual case records will be reviewed for a minimum of six months after initial radical prostatectomy.

Data will be abstracted from medical records at hospitals, cancer centres and urologists’ private consulting rooms using previously established methods [33].

Hospital level factors will be collected from centrally held records including specialist cancer centre and size. Patient level factors will be collected from the medical and hospital records including: month and year of birth, comorbidities, stage of cancer, Gleason score, PSA level at diagnosis, country of birth and private health insurance status. Remoteness of residence and socio-economic status (SES) of the cases will be assigned using their postcode of residence and the ARIA [70] and SEIFA [71], respectively.

Hormone therapy, comorbidities, pre-diagnostic PSA levels, Gleason score, country of birth, and health insurance status are potential barriers to referral for radiotherapy.

### Study sample

The unit of study will be the participating multidisciplinary teams (MDT). Nine public hospital-based MDTs in NSW will participate. The hospitals are located in both metropolitan and regional areas. Approximately four to ten urologists will be included at each site.

### Data analysis

The primary analysis will be conducted at the individual patient level using a generalized estimating equations (GEE) approach to account for repeated outcome observations within clusters (urologists and MDTs). The dependent variable for this analysis will be referral to a radiation oncology service for adjuvant radiotherapy or enrolment into the RAVES trial (versus no referral) for each prostate cancer case. The exposure variable will be the intervention status (pre versus post) of the hospital at the time of the post-prostatectomy consultation. Other independent variables will be added to the model if they are shown to be independently associated with radiotherapy referral and/or their inclusion in the model changes the linear coefficient of the intervention effect by more than 20% in absolute value. Analysis to determine the extent to which changes in urologists’ knowledge, attitudes and beliefs (Phase II) mediated any changes in referral patterns will be assessed by including clinicians’ change scores in the GEEs.

### Sample size and statistical power

Based on estimates from the NSW Central Cancer Registry and Medicare claims data we estimate that 3,517 NSW men will have a radical prostatectomy in 2013. Approximately 1,618 (46%) of these will be performed in the nine hospitals with urological MDTs participating in the ACI Urology Clinical Network according to linked cancer registry and hospital data for all NSW men diagnosed with prostate cancer. Assuming no major change has occurred in this distribution, there will be 1,348 radical prostatectomies over the 10 months of this trial. Of these, 20 to 50% or 270 to 671 men will be at ‘high risk’ [22-25]. The stepped wedge design is relatively insensitive to variations in the intracluster correlation (ICC) as a consequence of its efficient use of within-cluster and between-cluster information and has little impact on the study's power. However, based on the best available information, we estimate that the ICC for use of radiotherapy will be between 0.09 and 0.15 [72].

The most recently available data indicate 10% of high-risk men receive radiotherapy after surgery in NSW [33]. With the release of the Australian Cancer Network Clinical Practice Guidelines and the commencement of the RAVES trial we estimate that at the commencement of our trial, administration of radiotherapy following surgery will have increased to 15% to 20% of high-risk patients. Our stepped wedge study design with nine clusters, six time intervals (including the pre-intervention control step) and ICCs of 0.09 to 0.15 will have at least 80% power to detect an increase in referral to a radiation oncologist from 15% to 35%, or 20% to 40% if a minimum of 30% of patients are at high risk, and from 20% to 35% if at least 50% of prostate cancer cases are at high risk.

### Staff training and evaluation

Primary and secondary outcomes can be measured reliably through clinical data collection and this method has been used previously [33,73,74]. Research assistants conducting the medical record review will be trained and we will conduct a 10% blinded re-review to assess inter-rater reliability.

### Phase II: identify mechanisms of provider and organisational change

#### Design

‘Before and after’ mixed methods study to measure knowledge, attitudes, process, and explanatory variables.

### Urologists’ knowledge and attitudinal outcomes

#### Hypotheses

Compared with pre-intervention measures, urologists post-intervention will have: increased knowledge about the evidence for appropriate adjuvant radiotherapy for high-risk prostate cancer patients after radical prostatectomy and the associated risks and benefits of treatment; and more positive attitudes towards the need for referral to radiation oncology as a means to support fully-informed patient decision making.

### Data collection

A quantitative study of urologists will be conducted using a questionnaire to assess knowledge, beliefs, social influences, attitudes and motivation at three time points: baseline (pre-intervention); six months after the roll-out of the intervention; and at the end of the study (n ≈ 4 – 10 urologists per hospital). The survey is tailored to the intervention, uses previously identified domains (knowledge, beliefs, motivation, social influences), constructs, and generic questions to investigate the implementation of evidence-based practice [48], and is modelled on questions developed for other clinical conditions [75]. The measures using Likert scales have been developed through pilot testing and their feasibility and reliability will be assessed as part of the data collection in accordance with best practice [76]. Questions are consistent with those used in the baseline nationwide survey of urologists to enable comparison between groups. These surveys produce continuous scores for knowledge, beliefs, social influences, attitudes, and motivation at the clinician level that will be averaged for each hospital at each time point.

A follow up nationwide survey of urologist members of the Urological Society of Australia and New Zealand (USANZ) (n ≈ 370) will be conducted to determine whether urologists’ attitudes shifted locally/nationally without intervention.

### Process outcomes

#### Research question

Was the intervention implemented as intended?

### Data collection

The date of commencement of the intervention will be noted as the day the Urologist Clinical Leader within each site facilitated the educational intervention session. Agendas and minutes of subsequent MDT meetings will be reviewed using a method developed by members of the investigator team [77] to assess: numbers attending the meeting; frequency of mentioning the study; discussion of cases flagged by pathology; presentation of medical record review feedback; and changes in hospital practice as indicators of sustained interest in the intervention and organisational process changes.

### Research questions

1. Why did or did not the intervention result in evidence-based care?

2. Why was or was not the intervention implemented or sustained in hospitals?

### Data collection

1. Qualitative semi-structured interviews with Clinical Leaders at the end of the study to feedback study results and explore the reasons for them (n = 9).

2. Qualitative semi-structured telephone interviews, informed by feedback from Clinical Leaders, with urologists in the nine intervention hospitals at the end of the study to feedback study results and further explore the reasons for them (n ≈ 4 – 10 urologists per hospital).

### Data analysis

Survey data will be analysed using bivariable methods (means, t-tests and ANOVA for normally distributed continuous data; medians and non-parametric tests for non-normally distributed continuous data; and proportions and chi-squared tests for categorical data).

Semi-structured interview data will be analysed thematically using a matrix-based framework to organise data according to the theoretical framework used for the intervention design to identify why changes did or did not happen in the hospitals and why the intervention did or did not result in improved care.

### Research governance

The study has been approved by Royal Prince Alfred Research Ethics Committee (ID: X12-0388 & HREC/12/RPAH/584). Site-specific approval (SSAs) from the research governance office at each of the nine participating hospitals has been obtained. Site-specific approval from Cancer Council NSW ethics committee has been granted to cover data collection, storage and analysis at Cancer Council NSW.

## Trial status

The intervention and data collection phase of the study commenced in December 2013.

## Discussion

Clinical networks such as those established by the NSW Agency for Clinical Innovation are increasingly being viewed as an important strategy for increasing evidence-based practice in Australia and other countries. This interest in clinical networks is accompanied by significant investment in them but few studies have directly tested their effectiveness in driving implementation initiatives. To the authors’ knowledge, this study will be one of the first randomised controlled trials to test the effectiveness of clinical networks to lead changes in clinical practice in hospitals treating patients with high-risk cancer and improve evidence-based care.

### Limitations

The aim of this study is to target referral patterns of practising clinicians using the leverage of a clinical network. Intervention components therefore focus on the attitudinal and systems barriers at the urologist and hospital level. While we have sought consumer input into the design of provider-focused materials to provide guidance on what information patients want from consultation with their physician, ethics approval for the current study does not permit direct interaction with patients being treated by urologists in the study. The research team is developing a proposal for a sub-study focused on how patients can influence the treatment they receive, to be conducted at the end of Phase I.

## Abbreviations

ACI: NSW agency for clinical innovation; ARIA: Accessibility/remoteness index of Australia; CI: Chief investigator; CLICC: Clinician-led improvement in cancer care; GEE: Generalised estimating equation; HREC: Human research ethics committee; ICC: Intracluster correlation; MDT: Multidisciplinary team; MRN: Medical record number; NHMRC: National health and medical research council; NSW: New South Wales; PSA: Prostate specific antigen; RAVES: Radiotherapy adjuvant vs early salvage (phase iii randomised trial); SEIFA: Socio-economic indexes for areas; SES: Socioeconomic status; SSA: Site-specific approval; USANZ: Urological Society of Australia and New Zealand.

## Competing interests

Miranda Xhilaga is employed by the Prostate Cancer Foundation of Australia (PCFA), which has provided funds to support this research as part of the National Health and Medical Research Council (NHMRC) of Australia’s partnership project grant scheme (ID: 1011474). Andrew Brooks is the Co-Chair of the NSW Agency for Clinical Innovation (ACI) Urology Clinical Network and Mary Haines is on the Research Sub-Committee of the Agency for Clinical Innovation Board. This Agency has provided in-kind funds as part of the National Health and Medical Research Council (NHMRC) of Australia’s partnership project grant scheme (ID: 1011474). The contents of this paper are solely the responsibility of the individual authors and do not reflect the views of the National Health and Medical Research Council of Australia, Prostate Cancer Foundation of Australia or NSW Agency for Clinical Innovation. The other authors declare that they have no competing interests.

## Author’s contributions

The authors are the chief, associate, and honorary investigators of the research grant funding this research activity. BB, in collaboration with all other authors, conceptualised the research project and developed the protocol presented in this paper. All authors provided input into various aspects of the study, provided ongoing critique, and approved the final version of the manuscript.
